# Evaluation of a multiparametric MRI radiomic-based approach for stratification of equivocal PI-RADS 3 and upgraded PI-RADS 4 prostatic lesions

**DOI:** 10.1038/s41598-020-80749-5

**Published:** 2021-01-12

**Authors:** Valentina Brancato, Marco Aiello, Luca Basso, Serena Monti, Luigi Palumbo, Giuseppe Di Costanzo, Marco Salvatore, Alfonso Ragozzino, Carlo Cavaliere

**Affiliations:** 1 IRCCS SDN, Naples, Italy; 2 Department of Radiology, S. Maria Delle Grazie Hospital, Pozzuoli, Italy; 3 Institute of Biostructures and Bioimaging, National Research Council, Naples, Italy

**Keywords:** Urological cancer, Prostate cancer, Diagnostic markers, Predictive markers, Cancer, Medical research

## Abstract

Despite the key-role of the Prostate Imaging and Reporting and Data System (PI-RADS) in the diagnosis and characterization of prostate cancer (PCa), this system remains to be affected by several limitations, primarily associated with the interpretation of equivocal PI-RADS 3 lesions and with the debated role of Dynamic Contrast Enhanced-Magnetic Resonance Imaging (DCE-MRI), which is only used to upgrade peripheral PI-RADS category 3 lesions to PI-RADS category 4 if enhancement is focal. We aimed at investigating the usefulness of radiomics for detection of PCa lesions (Gleason Score ≥ 6) in PI-RADS 3 lesions and in peripheral PI-RADS 3 upgraded to PI-RADS 4 lesions (upPI-RADS 4). Multiparametric MRI (mpMRI) data of patients who underwent prostatic mpMRI between April 2013 and September 2018 were retrospectively evaluated. Biopsy results were used as gold standard. PI-RADS 3 and PI-RADS 4 lesions were re-scored according to the PI-RADS v2.1 before and after DCE-MRI evaluation. Radiomic features were extracted from T2-weighted MRI (T2), Apparent diffusion Coefficient (ADC) map and DCE-MRI subtracted images using PyRadiomics. Feature selection was performed using Wilcoxon-ranksum test and Minimum Redundancy Maximum Relevance (mRMR). Predictive models were constructed for PCa detection in PI-RADS 3 and upPI-RADS 4 lesions using at each step an imbalance-adjusted bootstrap resampling (IABR) on 1000 samples. 41 PI-RADS 3 and 32 upPI-RADS 4 lesions were analyzed. Among 293 radiomic features, the top selected features derived from T2 and ADC. For PI-RADS 3 stratification, second order model showed higher performances (Area Under the Receiver Operating Characteristic Curve—AUC— = 80%), while for upPI-RADS 4 stratification, first order model showed higher performances respect to superior order models (AUC = 89%). Our results support the significant role of T2 and ADC radiomic features for PCa detection in lesions scored as PI-RADS 3 and upPI-RADS 4. Radiomics models showed high diagnostic efficacy in classify PI-RADS 3 and upPI-RADS 4 lesions, outperforming PI-RADS v2.1 performance.

## Introduction

Prostate cancer (PCa) is one of the most commonly diagnosed malignant neoplasms among men^1^. Multiparametric Magnetic Resonance Imaging (mpMRI) has gradually gained in importance for both a timely diagnosis and an accurate characterization of PCa lesions, which play a key-role in all PCa patient management steps^2,3^.

With the goal of standardizing the acquisition and reporting of prostatic mpMRI imaging examinations, the European Society of Urogenital Radiology (ESUR) developed the Prostate Imaging-Reporting and Data System (PI-RADS) in 2013 and then updated it in 2015 (PI-RADS v2) and 2019 (PI-RADS v2.1)^4^.

PI-RADS evaluation is based on a 5-point scale associated with the probability that a combination of findings on mpMRI modalities (namely T2-weighted—T2, Diffusion Weighted MRI—DWI and Dynamic Contrast-Enhanced MRI—DCE-MRI, abbreviated) correlates with the presence of a clinically significant cancer for detected prostatic lesion. PI-RADS score ranges between 1 and 5, respectively indicating a very low and a very high likelihood that a lesion is malignant. The PI-RADS classification had a crucial role in PCa management since its development, and has proven to be a powerful tool for the identification and aggressiveness characterization of prostatic lesions^5–8^.

Although encouraging results have been reported in the literature on the role of the PI-RADS in the diagnosis and characterization of PCa, this system remains to be affected by several limitations, primarily associated with the interpretation of PI-RADS category 3 lesions, namely those lesions on prostate MRI that are termed as ‘intermediate’ or ‘equivocal on the presence of clinically significant cancer’^4^.

Despite the increase in diagnostic accuracy, reproducibility and score assignment easing of PI-RADS v2 and v2.1 compared to the first version, limitations related to risk stratification of PI-RADS 3 lesions still remain, since guidelines do not say how to deal with such imaging findings that are indeterminate^4,9,10^. PI-RADS 3 lesions present a challenge to treating urologists who must decide the optimal management option, namely whether to monitor with follow-up prostate-specific antigen (PSA) testing and imaging, or schedule immediate biopsy. An accurate and calibrated patient selection for prostate biopsy is essential in order to avoid unnecessary biopsies^9^. This equivocal imaging characterization may lead to high variability in practice patterns, costs, and potentially clinical outcomes across different institutions. Studies aimed at stratifying PI-RADS 3 lesions are limited^9,11–15^. Another limitation directly affecting PI-RADS 3 lesion assignment concerns the role of DCE-MRI, which is still regarded as very controversial and debated, and its added value in combination with T2 and DWI was still not clearly assessed. Currently, according to the new PI-RADS v2.1, DCE-MRI is only used to upgrade PI-RADS category 3 lesions to PI-RADS category 4, but only for lesions located in peripheral zone (PZ)^4^. However, the diagnostic value of DCE-MRI in prostate MRI remains subject of the current debate and it must be shown how many additional cases of PCa are found in PI-RADS 3 upgraded to PI-RADS 4 lesions. Radiomics, the extraction of multiple quantitative imaging features from medical images, represents an attractive tool which could overcome the clinical challenge associated with radiologist uncertainties related to PI-RADS 3 lesions and PI-RADS 3 upgraded to PI-RADS 4 lesions. Radiomic tool has been widely explored in the field of PCa and led to promising results, but especially in studies aiming at differentiating between normal and cancerous prostatic tissue, characterizing PCa lesions in terms of aggressiveness according to Gleason Score (GS), and also comparing diagnostic power of radiomic features with that of PI-RADS scoring^16–22^. However, to our knowledge, only Giambelluca et al.^23^ applied radiomic approach to stratify PI-RADS 3 lesions, and there are any studies aiming at investigating the power of radiomics in stratify PI-RADS 3 upgraded to PI-RADS 4.

In this context, we aimed at investigating the usefulness of radiomics for detection of PCa (GS ≥ 6) in PI-RADS category 3 lesions and in PI-RADS 3 upgraded to PI-RADS 4 lesions (upPI-RADS 4) in PZ.

## Methods and materials

### Patient population

We performed retrospective analysis of all mpMRI data of patients who underwent mpMRI of the prostate between April 2013 and September 2018 due to elevated PSA level and/or clinical suspicion of PCa and subsequently biopsy. mpMRI images and histopathology records were collected at H.S. Maria delle Grazie, Italy and informed consent was given before Magnetic Resonance (MR) examination^24^. The study was conducted in accordance with the Declaration of Helsinki, and the study protocol was approved by the Ethics Committee of the Istituto Nazionale Tumouri “Fondazione G. Pascale (protocol number 1/20). PI-RADS 1, 2 and 5 lesions were excluded. Examinations where a PI-RADS 3 lesion was present together with PI-RADS 4 or 5 lesion were excluded. Biopsy results were used as gold standard.

### MRI protocol

Routine clinical mpMRI acquisition includes T2, DCE-MRI, and DWI. The DWI includes an apparent diffusion coefficient (ADC) map generated at the time of acquisition. Patients were injected with contrast agent Gadoteridol (Gd-HP-DO3A; Pro Hance, Bracco Diagnostics, Princeton, NJ, USA) with a dose of 0.1 mL/kg before DCE-MRI acquisition. All patients were imaged using MAGNETOM-Avanto scanner (Siemens Healthcare, Erlangen, Germany) at 1.5 T with both endorectal coil and phase-array pelvic coil^24^. More details on the technical parameters of the MRI sequences are shown in Supplementary Table S1.

### PI-RADS assignment

After exclusion of PI-RADS 1, 2 and 5, imaging findings of the remaining patients were re-scored according the PI-RADS v2.1 by two radiologists, respectively with 10 and 8 years of experience. They independently reviewed MR images blinded to the results of biopsy and any clinical information. Evaluation of MR images was performed in two reading-sessions, following a similar procedure performed in our previous study^24^: the first reading-session was performed considering a biparametric MRI (bpMRI) protocol consisting of axial, sagittal and coronal T2 images and axial DWI images with their corresponding ADC maps and a b-computed image with b = 1400 s/mm^2^; the second reading-session was performed after 2 weeks considering the entire mpMRI protocol, and rearranging patient IDs in a different order, to reduce memory bias. Discrepancies were resolved by consensus. For both reading sessions, we assessed the inter-reader agreement between the two radiologists based on the ratings assigned before the consensus using the weighted Cohen’s kappa (*κ*) with linear weights^25–27^. The strength of agreement was evaluated as excellent if *κ* = 0.81–1.00, good if *κ* = 0.61–0.80, moderate if *κ* = 0.41–0.60, fair if *κ* = 0.21–0.40, and poor if *κ* = 0–0.20. After reaching consensus, PI-RADS 3 scored lesions after mpMRI evaluation and PI-RADS 3 lesions upgraded to PI-RADS 4 after DCE-MRI evaluation were selected.

### Biopsy protocol

All prostatic biopsies were TRUS-guided and performed using an 18-gauge tru-cut needle, under anesthesia. Each patient underwent both systematic biopsies, with an average of 12 random samples of the entire prostate gland, and target biopsy, with at least three samples taken from each lesion identified by MRI. The number of randomly-taken samples could vary depending on dimensions of prostate gland, as well as number of target samples could do depending on dimension of each lesion. Target sampling was performed with an MRI/TRUS fusion, using alternately the cognitive technique or dedicated software, coupled with ultrasound platforms from various companies^24^. The gross description included number and core lengths of needle biopsies. The specimens were fixed in buffered 10% formalin, and routinely processed. Thin sections of fuor microns were cut and stained with hematoxylin and eosin stain (H&E). Supplementary sections were performed for possible immunohistochemical stains to prove the loss of basal cells in small focus of cancer (p63, and high molecular weights keratin) combined with other antibodies overexpress in prostatic cancer (anti-AMACR/p504S). One senior pathologist (with more than 10 years of experience in prostate specimen interpretation) who was blinded to the MRI reports, reviewed pathological slices and classified tumors according to the 4th WHO classification, further grading them by Gleason scores and the group grade cancer^28–30^. The final report also included tumor extent in each needle biopsies and the percent core involvement by tumor.

### Image preprocessing and 3D ROI segmentation

ADC images were non-rigidly coregistered on T2 image using Elastix software (v. 4.9.0) in order to correct for typical spatial distortion arising from DWI acquisition. Subtraction DCE-MRI images were all resliced on T2 images. Two experienced radiologists were asked to consensually draw 3D regions of interest (ROIs) in the biopsied lesions with PI-RADS3 and upPI-RADS4, while also looking at the b = 1000 coregistered volume. Lesion segmentation was performed on T2 images using an in-house developed software for region labeling. During the segmentation procedure, the radiologists were blinded to both the histological results and all clinical information relative to the retrospective prostate mpMR images. Prior to radiomic features extraction, normalization was applied on T2 images intensities. Specifically, intensities were normalized by centering them at their respective mean value with standard deviation of all gray values in the original image^31–34^.

### Radiomic analysis

#### Feature extraction

Extraction of radiomic features from 3D ROIs on T2, registered ADC and resliced subtraction DCE-MRI images was performed using PyRadiomics, an open-source package for standardizing the extraction of radiomics data^32^. The extracted features can be classified into three classes: shape (n = 14), first-order statistics (n = 18), and second order textural statistics including grey level cooccurrence matrix—glcm—(n = 24), grey level run length matrix—glrlm—(n = 16), grey level size zone matrix—glszm—(n = 16), neighbouring gray tone difference matrix—ngtdm—(n = 5) and gray level dependence matrix—gldm—(n = 14). Detailed description and computing algorithms of the radiomic features were available at https://pyradiomics.readthedocs.io. The first and second order textural features were computed for T2, ADC and the DCE-MRI subtraction series with the highest mean signal intensity within the ROI^35,36^. Finally, a total of 293 features were extracted for each lesion, namely 14 shape features and, for each of the three MRI images (T2, ADC, DCE-MRI), 93 features including first- and second-order features. The full list of extracted radiomic features is provided in Supplementary Table S2. Two classification tasks were investigated: PCa/non-PCa PI-RADS3 and PCa/non-PCa upPI-RADS 4. So, procedures described in the following paragraphs are to be considered per classification task.

#### Feature selection

Feature normalization was performed before feature selection by using z normalization. Specifically, each feature was normalized as $z=(x-\stackrel{-}{x})/s$, where $x$, $\stackrel{-}{x}$, and $s$ are the feature, the mean, and the standard deviation, respectively^37,38^. Due to the relatively small patient sample size and high-dimensional feature size, we then performed feature selection process to select features most related to biopsy outcome, in order to construct prediction models. Feature selection was performed including two steps. In the first step the feature set was restricted through a univariate analysis by using nonparametric Wilcoxon rank-sum test performed to investigate their statistical significance with respect to the outcome (PCa vs non-PCa). The significantly different features (p < 0.05) were then selected and further reduced in the second step using Minimum Redundancy Maximum Relevance (mRMR) algorithm. mRMR algorithm selects an optimal set of features considering both the relevance for outcome prediction and the redundancy between features, using mutual information (MI) to measure both the relevance and the redundancy. At each step of mRMR feature selection process, the feature with the highest predictor importance score (defined as the difference between MI between outcome and the considered feature and the average MI of previously selected feature and the considered feature) will be added to the selected feature set^39,40^. The top five features with highest predictor importance score were finally used to construct radiomics prediction models. Feature selection procedures were implemented in MATLAB R2019b (The MathWorks Inc., Natick, MA, USA).

#### Multivariable model building and analysis

For each classification task, the reduced feature set was used to build logistic regression models of order from 1 to 5 that would best predict the presence of PCa using an imbalanced-adjusted bootstrap resampling (IABR) approach on 1000 bootstrap samples^41^. Specifically, 1000 bootstrap samples were randomly drawn with replacement from the available dataset and used as training set, while instances that do not appear in the bootstrap sample are the testing set^42^. Then, the imbalance-adjustment step was applied duplicating the number of positive instances by a factor equal to the number of negative instances, and the number of negative instances by a factor equal to the number of positive instances. This operation made the probability of picking a positive and a negative instance in the bootstrap sample the same^41^.

For each model order, the combination of features maximizing the 0.632+ area under the receiver operating characteristic curve (AUC) within 1000 bootstrap training and testing samples was identified^43,44^. Once optimal combination of features was identified for model orders 1–5, IABR on 1000 samples was performed again for all models in order to evaluate prediction performances.

## Results

### Patients characteristics

**Figure 1:**
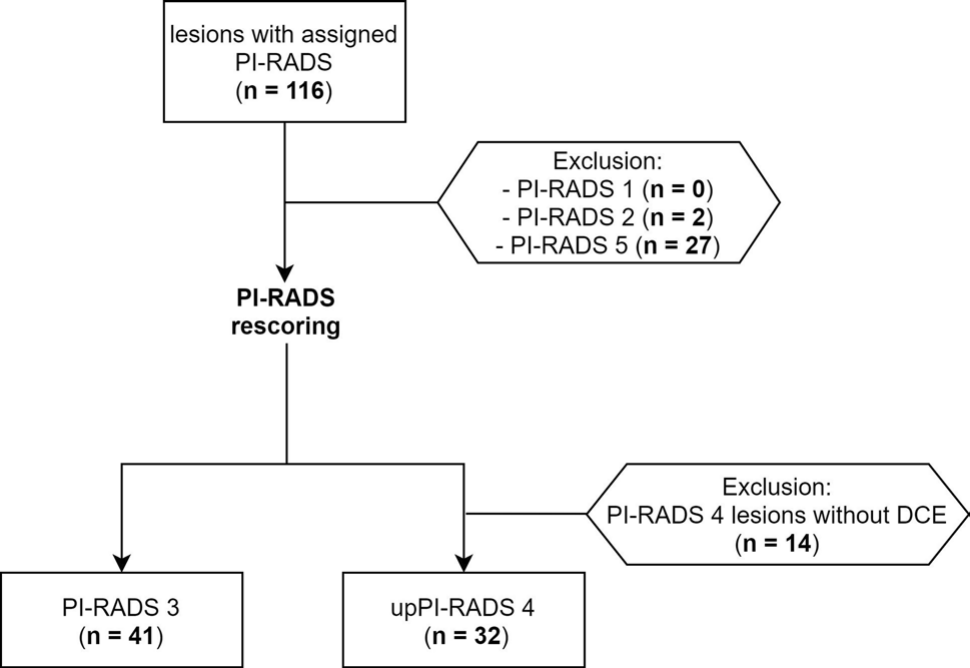
Flowchart showing how final cohort was selected. *PI-RADS* prostate imaging reporting and data system, *upPI-RADS 4* lesions scored as PI-RADS 4 after mpMRI PI-RADS assignment but evaluated as PI-RADS 3 after biparametric MRI reading session.

**Figure 2:**
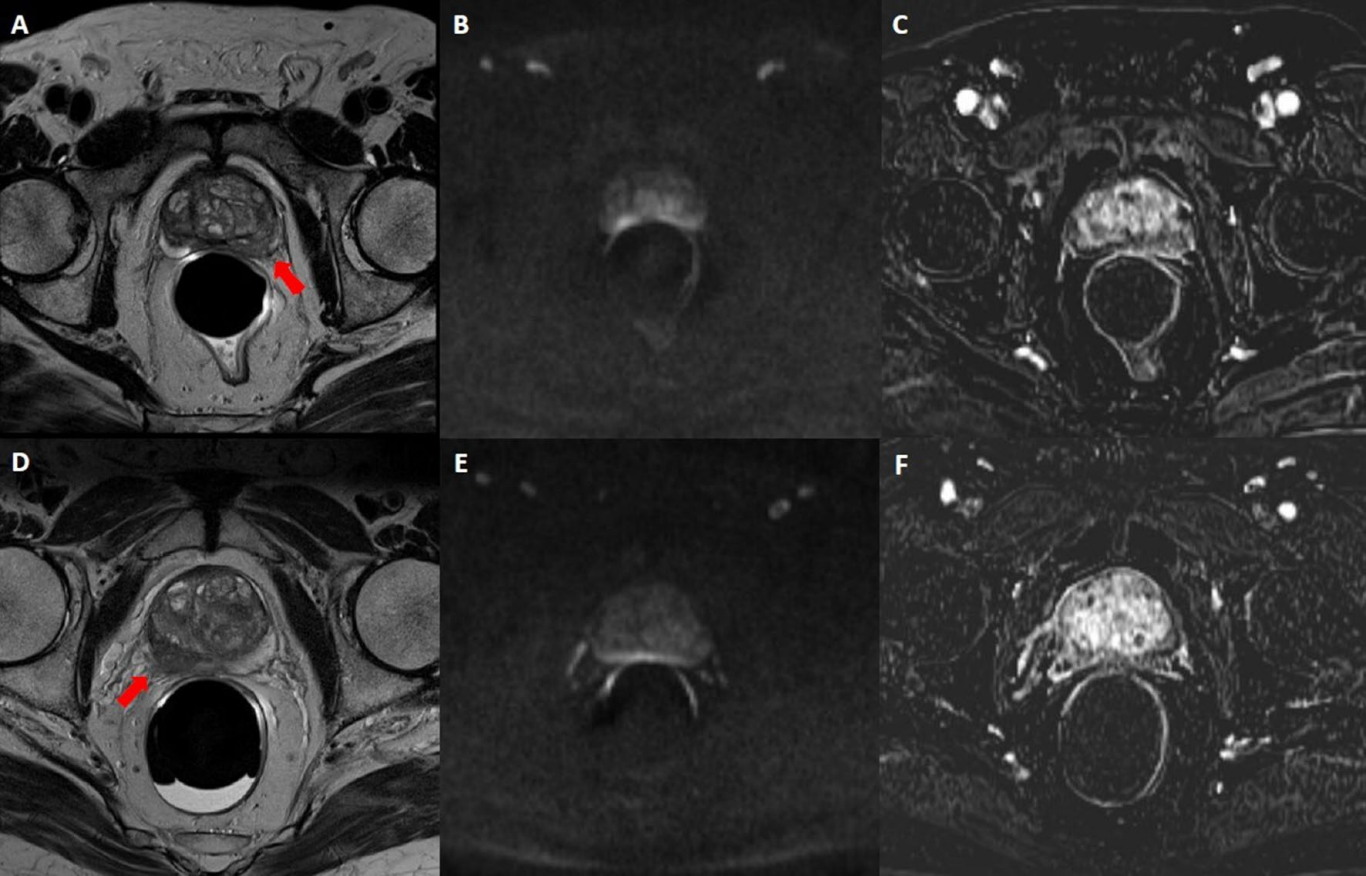
PI-RADS 3 scored alterations. In the first row, an example of PI-RADS 3 lesion confirmed at biopsy as GS 3 + 3: mpMRI showed reduced T2 signal intensity in the left PZ (**A**), altered diffusivity at b1000 DWI (**B**) without contrast enhancement (**C**). In the second row, an example of PI-RADS 3 lesion not confirmed at biopsy: mpMRI showed reduced T2 signal intensity in the right PZ (**D**), altered diffusivity at b1000 DWI (**E**) without contrast enhancement (**F**). *PI-RADS* prostate imaging-reporting and data system, *mpMRI* multiparametric MRI, *DWI* diffusion weighted MRI, *PZ* peripheral zone.

**Figure 3:**
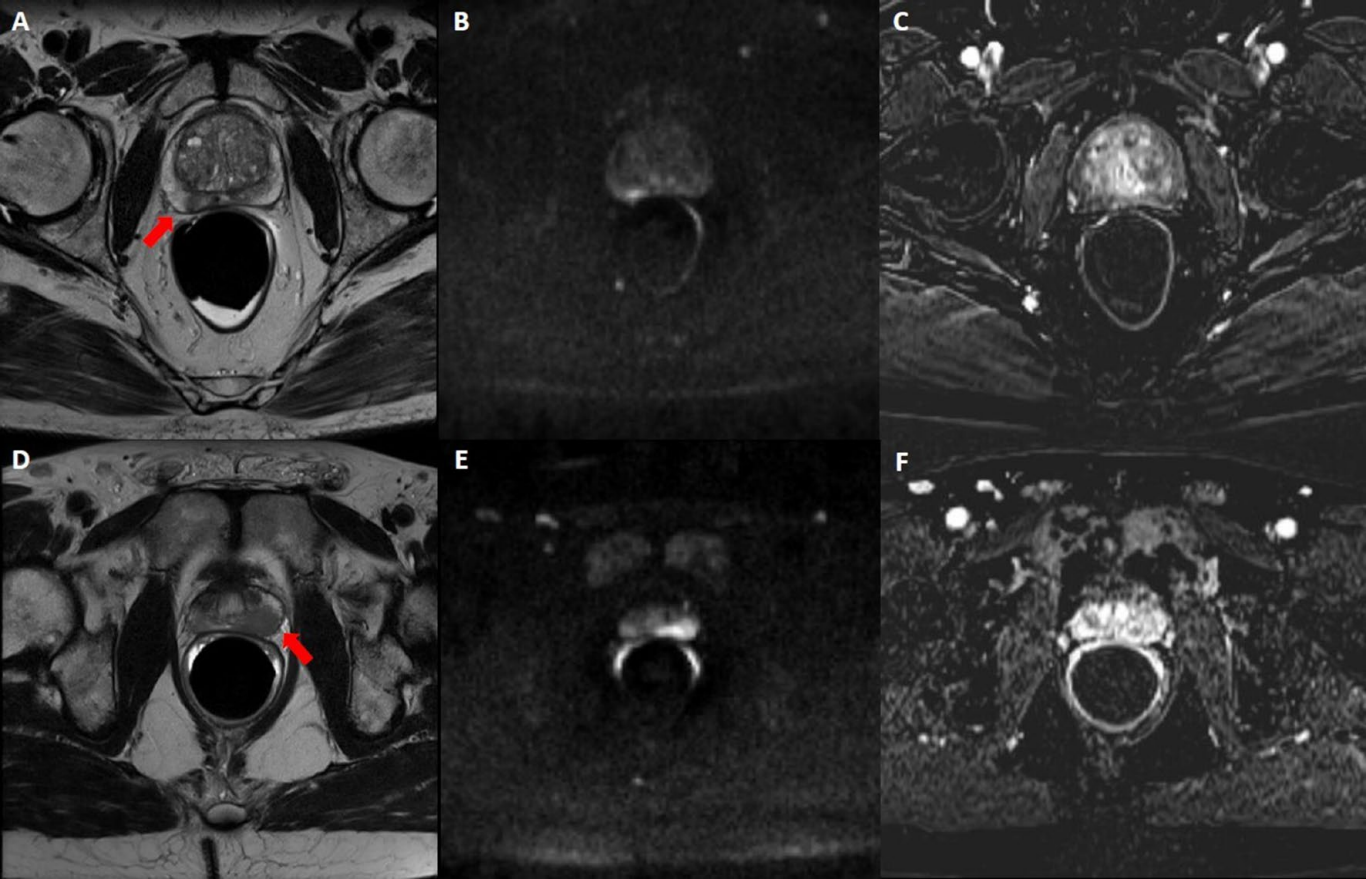
upPI-RADS 4 scored alterations. In the first row, an example of upPI-RADS 4 lesion confirmed at biopsy as GS 4 + 4: mpMRI showed reduced T2 signal intensity in the right PZ (**A**), altered diffusivity at b1000 DWI (**B**) with focal contrast enhancement (**C**). In the second row, an example of upPI-RADS 4 lesion classified as inflammatory at biopsy: mpMRI showed reduced T2 signal intensity in the right PZ (**D**), altered diffusivity at b1000 DWI (**E**) with contrast enhancement (**F**). *PI-RADS* prostate imaging-reporting and data system, *mpMRI* multiparametric MRI, *DWI* diffusion weighted MRI, *PZ* peripheral zone.

116 PCa lesions were finally identified. Among patients with double lesions, no one presented PI-RADS 3 lesion together with PI-RADS 4 or 5 lesion. 41 lesions were scored as PI-RADS 3 and 46 as PI-RADS 4. For lesions evaluated as PI-RADS 3, PI-RADS reevaluation confirmed a PI-RADS 3 score in both bpMRI and mpMRI reading session. For lesions evaluated as PI-RADS 4, PI-RADS reevaluation confirmed a PI-RADS 4 score in mpMRI reading session. Among these, 32 lesions were scored as PI-RADS 3 in bpMRI reading session, but were upgraded to 4 due to focal, positive DCE-MRI. The remaining 14 PI-RADS 4 lesions were excluded from our analysis. Finally, our study results were based on 41 PI-RADS 3 and 32 upPI-RADS 4 lesions. See Fig. 1 for the patient selection flow chart and Table 1 for characteristics of included patients. Inter-reader agreement based on the ratings assigned before the consensus was excellent (κ > 0.8) for both sessions (See Supplementary Section S3). Of selected PI-RADS 3 and upPI-RADS 4 lesions, 26 and 24 were positive for PCa, respectively. Examples of PI-RADS 3 and upPI-RADS 4 PCa and non-PCa lesions are shown in Figs. 2 and 3.

**Table 1:** Characteristics of included patients.

Variable	PI-RADS 3	upPI-RADS 4
**Clinical variables**		
No. lesions [n]	41	32
Median age [years (range)]	66 (54 – 78)	68 (54 – 80)
Mean PSA level (ng/mL)	10.36	8.56
Prostate volume (cm^3^)	55.76	55.6
Prostatic zone		
PZ [n (%)]	35 (85.4)	32 (100)
TZ [n (%)]	2 (4.9)	0 (0)
CG [n (%)]	4 (9.7)	0 (0)
**Biopsy results**		
PCa negative [n (%)]	15 (36.58)	8 (25)
PCa positive [n (%)]	26 (63.42)	24 (75)
Gleason scores for PCa lesions [n (%)]		
3 + 3	15 (57.7)	13 (52)
3 + 4	3 (11.5)	2 (8)
4 + 3	5 (19.3)	6 (24)
4 + 4	3 (11.5)	4 (16)

*PSA* prostate-specific antigene, *PCa* prostate cancer, *PZ* peripheral zone, *TZ* transition zone, *CG* central gland, *PI-RADS* prostate imaging-reporting and data system.

### Radiomic analysis

**Figure 4:**
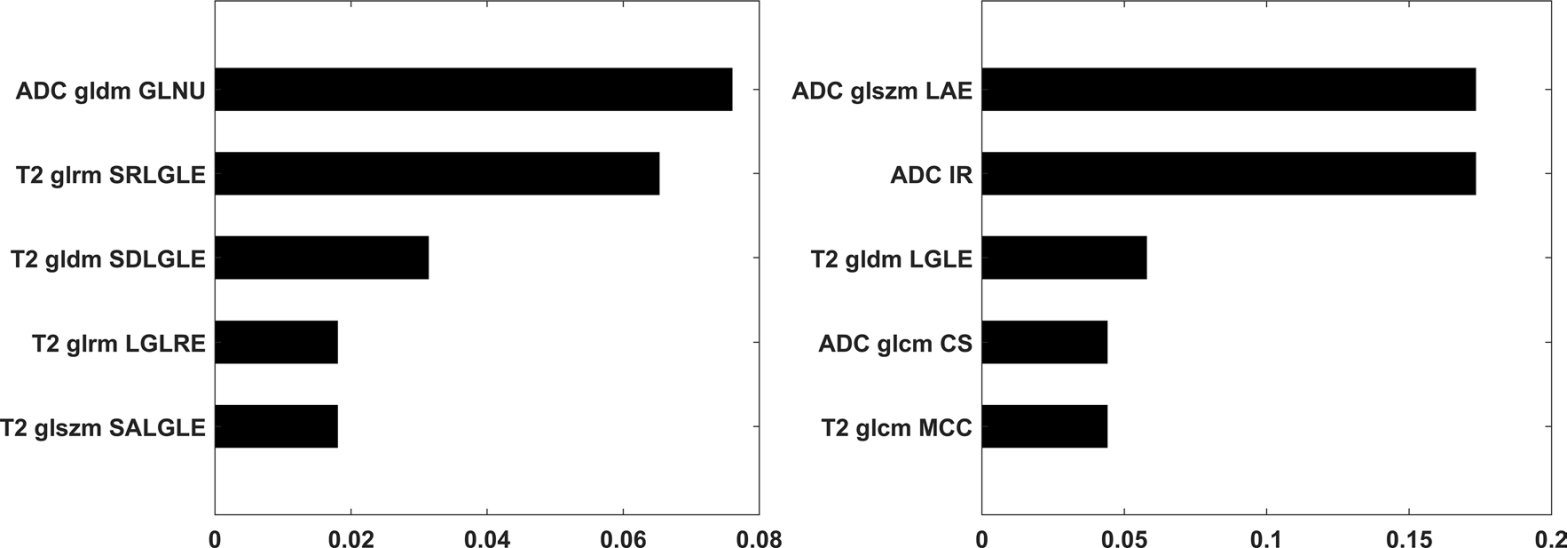
Bar plots of radiomic features ranked according to their relevance-redundancy predictor importance score for PI-RADS 3 (left side) upPI-RADS4 (right side) lesion detection tasks. *ADC* apparent diffusion coefficient, *glcm* gray level co-occurrence matrix, *glrlm* grey level run length matrix, *gldm* gray level dependence matrix, *glszm* grey level size zone matrix, *SDLGLE* Small Dependence Low Gray Level Emphasis, *SALGLE* Small Area Low Gray Level Emphasis; *SRLGLE* Short Run Low Gray Level Emphasis; *LAE* Large Area Emphasis; *IR* Interquartile Range; *LGLE* Low Gray Level Emphasis; *CS* Cluster Shade; *MCC* Maximal Correlation Coefficient.

Univariate analysis revealed 36 and 43 statistically significant features, respectively for PI-RADS3 and upPI-RADS 4 classification tasks. Statistically significant features are reported in Supplementary Tables S4 and S5. By using the mRMR method on these features, the five highest mRMR-ranked features were selected to build the prediction models. Bar plots of predictor importance score for the top five features selected by mRMR for each classification task are shown in Fig. 4.

**Figure 5:**
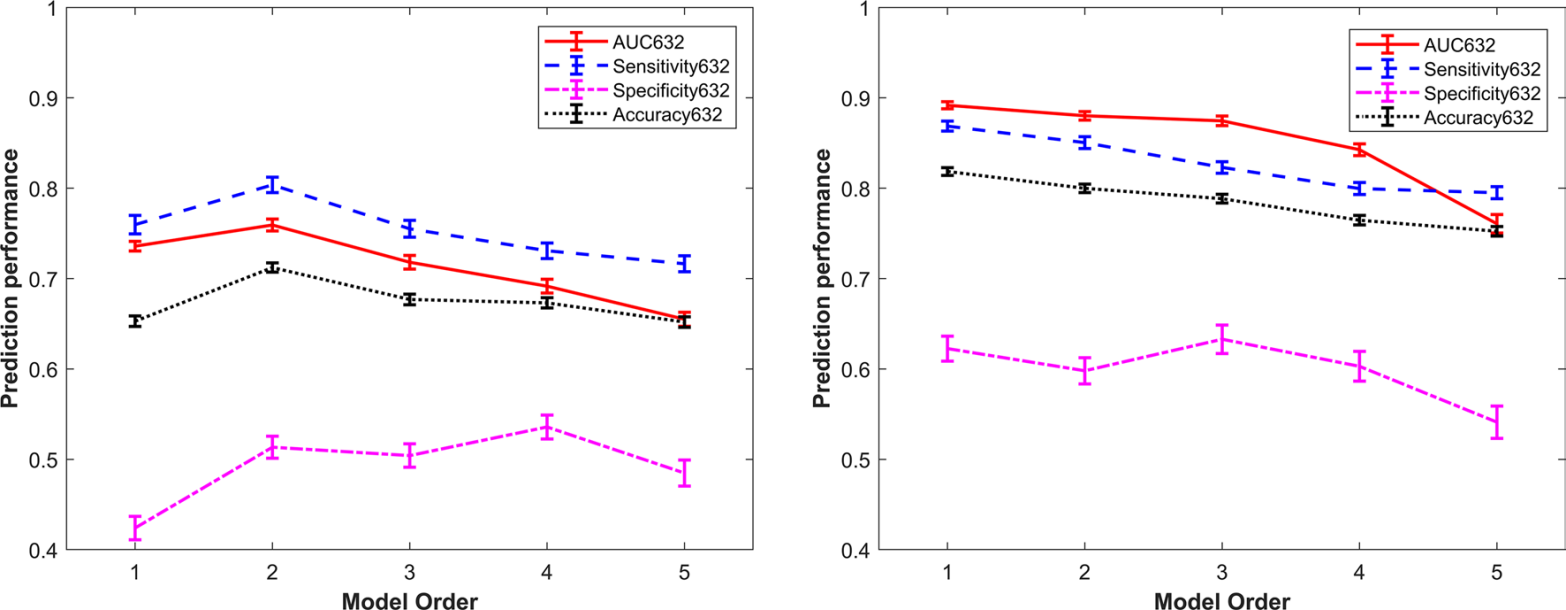
Prediction performances of models from order 1 to 5 for detection of PCa in PI-RADS 3 lesions (on the left) and in upPI-RADS 4 lesions (on the right).

Multivariable logistic regression models of order from 1 to 5 were obtained, and their prediction performance for the two classification tasks were reported in Supplementary Table S6 and showed in Fig. 5. By inspecting curves in Fig. 5 and values in Supplementary Table S6, we determined that the simplest multivariable model with the best prediction performances were reached by second order model for PI-RADS 3 and for first and third upPI-RADS4 classification task. For PI-RADS 3 lesion detection, second order model was chosen due to a slightly higher mean sensitivity, specificity and accuracy (80%, 51%, 71%, respectively) respect to first order model (76%, 42%, 65%, respectively), which showed comparable AUC (AUC = 74% for first order model, AUC = 0.76 for second order model). For models of order from 3 to 5, prediction performances get worse. For upPI-RADS 4 classification task, first order model showed higher performances (AUC = 89%, sensitivity = 87%, specificity = 62%, accuracy = 82%) respect to higher order models. However, promising results were also obtained from third order model performance metrics.

## Discussions

The aim of our study was to investigate whether an mpMRI-based radiomic approach was able to outperform PI-RADS v2.1 performance in stratifying PI-RADS 3 and PI-RADS 3 upPI-RADS4 lesions, which are directly related to still opening challenges and controversies for PI-RADS score assignment. In particular, concerning PI-RADS3 lesions, indicating an equivocal likelihood of significant PCa, the major clinical challenge is to avoid excessive biopsies and simultaneously improve PCa detection^10,14^. On the other hand, the upgrade of upPI-RADS 4 lesions depends on DCE-MRI, its role in prostate mpMRI remains debated for PCa detection^45,46^. On the basis of promising results offered by radiomic approach for PCa detection, characterization and assessment in comparison with PI-RADS assignment, we investigated the power of radiomics for PI-RADS 3 and upPI-RADS 4 stratification, which, despite its relevancy for the previous mentioned reasons, still represents a little-explored field. Therefore, using biopsy as reference standard, we tested two classification tasks aiming at detecting PCa (namely lesions with GS ≥ 6) in PI-RADS 3 and upPI-RADS 4 scored lesions, respectively. Prediction models performances varied depending on the classification task and the model order. The obtained results for PCa positive PI-RADS 3 detection showed that the most relevant features for this classification task were texture features arising from T2 and ADC. However, the best multivariable prediction model was those built using T2 glrlm SRLGLE and T2 glrlm LGLRE texture features. The obtained results for PCa positive upPI-RADS 4 detection showed that the most relevant features for this classification task were texture features arising from T2 and ADC and interquartile range of ADC value. However, the best multivariable prediction model was that built using ADC IR. Promising results were also obtained using third order model built using ADC IR together with glszm LAE and T2 glcm MCC texture features. Interestingly, for both classification tasks, specificity was found to be lower than the other performance parameters across each model order, highlighting that our models may be prone to false positive errors. Previous studies aimed at stratifying PI-RADS 3 lesions^9,11,13–15^, but only one investigated the power of radiomic features for this purpose^23^. Our results on PI-RADS 3 lesions are in line with those obtained in this work, in which authors also reported that T2 and ADC texture features could help in stratification of PI-RADS 3 lesions. However, several differences respect to our work should be highlighted. First, in the above-mentioned work, authors just analyzed texture features arising from T2 and ADC maps images, omitting analysis of shape and first order features. In our work we also analyzed these features, even if none of them were useful for building predictive models. Second, we also evaluated features associated with DCE-MRI. However, none of them were useful for building predictive models. These results suggest that the inclusion of radiomic features derived from DCE-MRI does not provide a clear added value for PCa detection of PI-RADS 3 lesions. On one hand, this would justify the choice to exclude DCE-MRI from dominant sequences for PI-RADSv2 and PI-RADS v2.1 score assessment^17,24,45^. Similar considerations also apply to upPI-RADS 4 classification task, for which DCE-MRI features did not even survive in the univariate analysis preceding the mRMR step. However, a direct comparison with studies applying radiomic analysis restricted to this kind of lesions could not be applied, since literature is lacking in this field to our knowledge. Ullrich et al.^46^ discussed about the added value of DCE-MRI in a large cohort of patients with assigned PI-RADS 4 in a recent study, finding DCE-MRI useful to avoid underestimation and misclassification of clinically significant PCa and improve detection rates in PI-RADS 4 patients.

Strengths of our study were that we use PI-RADS v2.1 assessment, which should not affect the overall diagnostic accuracy when compared to PI-RADS v2, but should improve inter-reader variability and simplify score assignment^4,24,45^. Moreover, the use of 3D ROI should reduce inter-reader variability by avoiding the need to select a single-slice corresponding to a portion of a lesion and allow for a more complete description of the lesion given by the increasing of the number of points considered for features computation, which should improve the accuracy of characterization of heterogeneous lesions and reduce sampling errors^47,48^. Then, as recommended by the IBSI (Image Biomarker Standardization Initiative) guidelines, we normalized raw images to account for the variable intensity ranges of MRI data (acquired in arbitrary units) and improve robustness of radiomics features^34,49–52^. Lastly, a special attention should be given to the potential clinical impact of our work that lies in the fact that we attempted to overcome the clinical challenge associated with radiologist uncertainties related to PI-RADS 3 lesions, as well as those related to upPI-RADS 4 lesions due to the previously mentioned reasons^10,45,53^.

Despite the above, this study suffers from some limitations. First, PI-RADS 3 and upPI-RADS 4 datasets were too small and imbalanced to generalize results. A larger and more balanced study group is needed to better conduct a radiomic analysis and build more robust prediction models. In particular, although the IABR strategy we used for model building is a common reliable approach in case of small and imbalanced datasets, a larger sample size would allow to use part of the dataset for the training, and part for testing and validating the performance of the classifier with external datasets^44,54,55^. Given the small sample size for both PI-RADS 3 and upPI-RADS 4 groups, we did not perform a separate analysis aiming at detecting clinically significant PCa lesions (namely GS ≥ 7). Moreover, in PI-RADS 3 analysis, the low number of lesions prevent us to perform a sub-analysis on the basis of prostatic zone, similar to analysis performed by Ginsburg et al. for PCa detection in histopathological proven lesion^56^. In addition, although features arising from DCE-MRI parameters proved not to be useful for building prediction models. Further analysis on original DCE-MRI images^57^ and/or maps of pharmacokinetic parameters^58^ may be investigated, helping to overcome controversies related to DCE-MRI and clarify its role in PCa management. It could be also interesting to investigate if performances of prediction models for PCa detection in PI-RADS 3 and upPI-RADS 4 lesions could improve adding features arising from advanced diffusion models prediction model, which were found to be promising for detection and characterization of PCa, even if their role is not clearly affirmed due to the lack of a standardized diffusion MRI protocol^17,59^. Another aspect to highlight is that also delineations of prostatic lesions are prone to inter-observer variability. In fact, it should be considered that we delineated only the prostatic lesion for feature extraction, and it is well-recognized that small volumes might lead to uncertainties in feature extraction^60–63^.

In conclusion, our preliminary results support the significant role of T2 and ADC radiomic features, mainly second-order texture features, for PCa detection in lesions scored as PI-RADS 3 and upPI-RADS 4.

## Supplementary Information

Supplementary Information.
