# Supplementary material for: Evaluation of a multiparametric MRI radiomic-based approach for stratification of equivocal PI-RADS 3 and upgraded PI-RADS 4 prostatic lesions
Source: Sci Rep. 2021 Jan 12;11:643. doi: 10.1038/s41598-020-80749-5 (PMC7804929; doi:10.1038/s41598-020-80749-5)
Supplement: Supplementary file 1 — Supplementary Information. [file 41598_2020_80749_MOESM1_ESM.pdf]

Evaluation of a multiparametric MRI radiomic-based approach for stratification of equivocal PI-RADS 3 and upgraded PI-RADS 4 prostatic lesions.

Valentina Brancato[1,*], Marco Aiello[1], Luca Basso[1], Serena Monti[3], Luigi Palumbo[2], Giuseppe Di Costanzo[2], Marco Salvatore[1], Alfonso Ragozzino[2], Carlo Cavaliere[1]

[1] IRCCS SDN, Naples, Italy
[2] Department of Radiology, S. Maria delle Grazie Hospital, Pozzuoli, Italy.
[3] Institute of Biostructures and Bioimaging, National Research Council, Naples, Italy
[*] corresponding author: valentina.brancato@synlab.it

Supplementary Materials

S1. MRI protocol

Sequence	TR (msec)	TE (msec)	ST (mm)	Avg.	BW	Matrix	FOV (mm)	FA
T2W Sagittal	4740	102	3	3	200	320×288	200×200	137
T2WAxial	5610	102	3	3	200	320×310	200×200	123
T2WCoronal	4000	102	3	2	200	320×310	200×200	138
T2WLymphnodes	4000	93	5.5	2	130	488×197	341×390	135
DWIa	4624	77	3	6	1698	128×128	250×250	90
T1 vibe tra FA	5.5	2.34	3.5	8	300	320×112	208×417	2
T1 vibe tra FA	5.5	2.34	3.5	8	300	320×112	208×417	15
T1 vibedynb	5.5	2.34	3.5	1	300	320×112	208×417	10

Table S1. *Parameters of mpMRI sequences. TR = Repetition Time; TE = Echo Time; ST = Slice Thickness; Avg. = Averages; BW = Bandwidth; FOV = Field of view; FA = Flip angle.*

[a] DWI performed with b-values of 50, 400 and 1000 s/mm2.

[b] with 32 measurements.

S2. Feature extraction

Feature Type	*Feature name*
**Shape**<br>**[n = 14]**	shape Flatness<br>shape Sphericity<br>Maximum2D Diameter Row<br>shape MeshVolume<br>shape Elongation<br>shape Maximum2DDiameterColumn<br>shape VoxelVolume<br>shape SurfaceArea<br>shape MinorAxisLength<br>shape Maximum3DDiameter<br>shape MajorAxisLength<br>shape LeastAxisLength<br>shape SurfaceVolumeRatio<br>shape Maximum2DDiameterSlice
**First Order**<br>**[n = 18]**	Skewness<br>Maximum<br>MeanAbsoluteDeviation<br>Kurtosis<br>Range<br>Energy<br>InterquartileRange<br>Variance<br>Mean<br>10Percentile<br>90Percentile<br>Uniformity<br>Median

	RobustMeanAbsoluteDeviation<br>RootMeanSquared<br>Minimum<br>TotalEnergy<br>Entropy
**Second order (GLCM)**<br>**[n = 24]**	glcm SumSquares<br>glcm Contrast<br>glcm InverseVariance<br>glcm ClusterTendency<br>glcm DifferenceAverage<br>glcm Idm<br>glcm ClusterShade<br>glcm Imc2<br>glcm Idn<br>glcm ClusterProminence<br>glcm Id<br>glcm Imc1<br>glcm Autocorrelation<br>glcm SumEntropy<br>glcm MaximumProbability<br>glcm MCC<br>glcm Correlation<br>glcm JointEntropy<br>glcm Idmn<br>glcm JointAverage<br>glcm SumAverage<br>glcm DifferenceEntropy<br>glcm DifferenceVariance<br>glcm JointEnergy
**Second Order (GLRLM)**<br>**[n = 16]**	glrlm ShortRunHighGrayLevelEmphasis<br>glrlm RunLengthNonUniformity<br>glrlm ShortRunEmphasis<br>glrlm LongRunLowGrayLevelEmphasis<br>glrlm RunPercentage<br>glrlm GrayLevelVariance<br>glrlm LowGrayLevelRunEmphasis<br>glrlm GrayLevelNonUniformity<br>glrlm RunVariance<br>glrlm LongRunHighGrayLevelEmphasis<br>glrlm ShortRunLowGrayLevelEmphasis<br>glrlm RunLengthNonUniformityNormalized<br>glrlm LongRunEmphasis<br>glrlm GrayLevelNonUniformityNormalized<br>glrlm HighGrayLevelRunEmphasis<br>glrlm RunEntropy
**Second Order (GLSZM)**<br>**[n = 16]**	glszm SizeZoneNonUniformity<br>glszm LargeAreaEmphasis<br>glszm SmallAreaEmphasis<br>glszm ZonePercentage<br>glszm GrayLevelVariance<br>glszm LargeAreaLowGrayLevelEmphasis<br>glszm GrayLevelNonUniformity<br>glszm SmallAreaHighGrayLevelEmphasis<br>glszm ZoneVariance<br>glszm LargeAreaHighGrayLevelEmphasis<br>glszm HighGrayLevelZoneEmphasis<br>glszm SizeZoneNonUniformityNormalized<br>glszm ZoneEntropy<br>glszm SmallAreaLowGrayLevelEmphasis<br>glszm GrayLevelNonUniformityNormalized<br>glszm LowGrayLevelZoneEmphasis
**Second Order (NGTDM)**<br>**[n = 5]**	ngtdm Coarseness<br>ngtdm Strength<br>ngtdm Busyness<br>ngtdm Complexity<br>ngtdm Contrast
**Second Order (GLDM)**<br>**[n = 14]**	gldm LargeDependenceLowGrayLevelEmphasis<br>gldm DependenceNonUniformity

	gldm SmallDependenceLowGrayLevelEmphasis<br>gldm LowGrayLevelEmphasis<br>gldm LargeDependenceHighGrayLevelEmphasis<br>gldm DependenceEntropy<br>gldm DependenceVariance<br>gldm GrayLevelVariance<br>gldm GrayLevelNonUniformity<br>gldm SmallDependenceEmphasis<br>gldm LargeDependenceEmphasis<br>gldm SmallDependenceHighGrayLevelEmphasis<br>gldm HighGrayLevelEmphasis<br>gldm DependenceNonUniformityNormalized

Table S2. *Extracted radiomic features. First (n=18) and second order features (n = 75) were extracted from T2, DWI and DCE images, for a total of 93 features for each of the three MRI images. For each lesion, a total of 293 radiomic features were extracted (14 shape features, 93 features including first- and second-order features from T2, 93 features including first- and second-order features from ADC, 93 features including first- and second-order features from DCE-MRI).*

S3. Interobserver agreement

Inter-reader agreement was excellent for both sessions (κ >0.8). Specifically, for biparametric MRI session κ = 0.86, while for multiparametric MRI session κ = 0.91. See Table S3. for 95% confidence interval lower and upper bounds for κ-value, as well as the agreement rate between the two readers (percentage of lesions in agreement, divided by the total number of lesions).

bpMRI session		***mpMRI session***	
κ [95% CI]	***AR, %***	***κ [95% CI]***	***AR, %***
0.86 [0.70-1.00]	96.5	0.91 [0.81-1.00]	95.4

Table S3. *Results of interobserver agreement for each reading session (bpMRI and mpMRI). Numbers in square brackets are 95% confidence interval lower and upper bounds for κ-value. Abbreviations: bpMRI = biparametric MRI; mpMRI = multiparametric MRI; κ = Cohen's kappa; CI = Confidence AR = Agreement Rate.*

S4. Univariate analysis

**Feature name**	**p-value**
shape Maximum 2D DiameterRow	0.026
Shape Mesh Volume	0.031
Shape Voxel Volume	0.029
Shape Surface Area	0.031
Shape Minor Axis Length	0.013
Shape Surface Volume Ratio	0.036
T2 firstorder Kurtosis	0.015
T2 firstorder Energy	0.015
T2 firstorder Total Energy	0.019
T2 glszm Low Gray Level Zone Emphasis	0.013
T2 glszm Gray Level Non Uniformity	0.047
T2 glszm Small Area Low Gray Level Emphasis	0.015
T2 glcm MCC	0.036
T2 glcm Idn	0.041
T2 glcm Idmn	0.05
T2 gldm Small Dependence Low Gray Level Emphasis	0.01
T2 gldm Gray Level Non Uniformity	0.047
T2 gldm Low Gray Level Emphasis	0.011
T2 gldm Large Dependence High Gray Level Emphasis	0.036
T2 glrlm Long Run Low Gray Level Emphasis	0.011
T2 glrlm Low Gray Level Run Emphasis	0.011
T2 glrlm Gray Level Non Uniformity	0.034
T2 glrlm Short Run Low Gray Level Emphasis	0.009
T2 glrlm Run Length Non Uniformity	0.05

ADC glrlm Run Length Non Uniformity	0.031
ADC glrlm Gray Level Non Uniformity	0.011
ADC ngtdm Strength	0.036
ADC glszm Gray Level Non Uniformity	0.011
ADC firstorder Energy	0.022
ADC firstorder Total Energy	0.026
ADC gldm Gray Level Non Uniformity	0.027
ADC gldm Dependence Non Uniformity Normalized	0.047
ADC glcm Imc1	0.026
DCE glrlm Gray Level Non Uniformity	0.029
DCE ngtdm Coarseness	0.038
DCE gldm Gray Level Non Uniformity	0.029

Table S4. *Univariate analysis results for PI-RADS 3 lesions. For each feature, p-values refers to Wilcoxon rank-sum test result between PCa PI-RADS 3 and non-PCa PI-RADS 3 lesions.*

**Feature name**	**p-value**
Shape Elongation	0.035
T2 glszm LowGrayLevelZoneEmphasis	0.043
T2 glcm MCC	0.028
T2 glcm Correlation	0.048
T2 glrlm Short Run Low Gray Level Emphasis	0.039
T2 glrlm Low Gray Level Run Emphasis	0.035
T2 glrlm Long Run Low Gray Level Emphasis	0.048
T2 gldm Low Gray Level Emphasis	0.035
ADC glrlm Gray Level Variance	0.004
ADC glrlm Gray Level Non Uniformity Normalized	0.012
ADC glrlm Run Entropy	0.02
ADC ngtdm Strength	0.014
ADC ngtdm Busyness	0.02
ADC glszm Large Area Emphasis	0.007
ADC glszm Zone Percentage	0.007
ADC glszm Gray Level Variance	0.022
ADC glszm Zone Variance	0.005
ADC glszm Gray Level Non Uniformity Normalized	0.025
ADC firstorder Mean Absolute Deviation	0.002
ADC firstorder Range	0.039
ADC firstorder Interquartile Range	0.001
ADC firstorder Variance	0.003
ADC firstorder 90 Percentile	0.043
ADC firstorder Uniformity	0.007
ADC firstorder Robust Mean Absolute Deviation	0.003
ADC firstorder Entropy	0.028
ADC gldm Dependence Variance	0.012
ADC gldm Gray Level Variance	0.003
ADC gldm Small Dependence Emphasis	0.02
ADC gldm Large Dependence Emphasis	0.011
ADC gldm Small Dependence High Gray Level Emphasis	0.031
ADC gldm Dependence Non Uniformity Normalized	0.01
ADC glcm Sum Squares	0.005
ADC glcm Cluster Tendency	0.004
ADC glcm Cluster Shade	0.043
ADC glcm Imc2	0.012
ADC glcm Cluster Prominence	0.007
ADC glcm Imc1	0.048

ADC glcm Autocorrelation	0.028
ADC glcm Sum Entropy	0.025
ADC glcm MCC	0.008
ADC glcm Joint Average	0.048
ADC glcm Sum Average	0.048

Table S5. *Univariate analysis results for PI-RADS 3 lesions. For each feature, p-values refers to Wilcoxon rank-sum test result between PCa upPI-RADS4 and non-PCa upPI-RADS4 lesions.*

S5. Prediction performance of classification tasks

	PI-RADS 3					upPI-RADS 4				
**Order**	**Features**	**AUC ± SE**	**Sensitivity ± SE**	**Specificity ± SE**	**Accuracy ± SE**	**Features**	**AUC ± SE**	**Sensitivity ± SE**	**Specificity ± SE**	**Accuracy ± SE**
1	T2 gldm SDLGLE	0.736 ± 0.005	0.76 ± 0.01	0.424 ± 0.013	0.653 ± 0.006	ADC IR	0.892 ± 0.004	0.869 ± 0.006	0.622 ± 0.014	0.818 ± 0.004
2	T2 glrlm SRLGLE; T2 glrlm LGLRE;	0.759 ± 0.007	0.804 ± 0.009	0.513 ± 0.012	0.712 ± 0.005	ADC IR; T2 glcm MCC	0.88 ± 0.005	0.85 ± 0.007	0.598 ± 0.014	0.8 ± 0.005
3	T2 glrlm SRLGLE; T2 glrlm LGLRE; ADC gldm GLNU	0.718 ± 0.008	0.755 ± 0.009	0.504 ± 0.013	0.677 ± 0.006	ADC LAE; ADC IR; T2 glcm MCC	0.875 ± 0.005	0.823 ± 0.006	0.633 ± 0.016	0.788 ± 0.005
4	ADC gldm GLNU; T2 gldm SDLGLE; T2 glrlm LGLRE; T2 glrlm SRLGLE	0.692 ± 0.008	0.731 ± 0.009	0.536 ± 0.013	0.673 ± 0.006	ADC LAE; ADC IR; T2 glcm MCC; T2 LGLE	0.843 ± 0.007	0.8 ± 0.007	0.603 ± 0.016	0.765 ± 0.005
5	ADC gldm GLNU; T2 gldm SDLGLE; T2 glrlm LGLRE; T2 glrlm SRLGLE; T2 glszm SALGLE	0.655 ± 0.008	0.716 ± 0.009	0.485 ± 0.014	0.652 ± 0.006	ADC LAE; ADC IR; T2 glcm MCC; T2 gldm LGLE; ADC CS	0.761 ± 0.01	0.795 ± 0.007	0.541 ± 0.018	0.752 ± 0.005

Table S6. *Results of multivariate analysis for PI-RADS 3 and upPI-RADS 4 lesions. For each model (from order 1 to 5), AUC, sensitivity, specificity and accuracy were reported with the standard error on a 95% confidence interval over all bootstrap sample. Abbreviations: PI-RADS = Prostate Imaging and Reporting and Data System; upPIRADS = upgraded PI-RADS; AUC = Area Under the ROC Curve; SE = Standard Error; ADC = Apparent Diffusion Coefficient; glcm = gray level co-occurrence matrix; glrlm = grey level run length matrix; gldm = gray level dependence matrix ; glszm = grey level size zone matrix; SDLGLE = Small Dependence Low Gray Level Emphasis; SALGLE = Small Area Low Gray Level Emphasis; SRLGLE = Short Run Low Gray Level Emphasis; LAE = Large Area Emphasis; IR = Interquartile Range; LGLE = Low Gray Level Emphasis; CS = Cluster Shade; MCC = Maximal Correlation Coefficient.*
